# Challenges in the translation and commercialization of cell therapies

**DOI:** 10.1186/s12896-015-0190-4

**Published:** 2015-08-07

**Authors:** Brittany P. Dodson, Aaron D. Levine

**Affiliations:** School of Public Policy, Georgia Institute of Technology, Atlanta, GA USA; School of Public Policy, Parker H. Petit Institute for Bioengineering and Bioscience, Georgia Institute of Technology, Atlanta, GA USA

## Abstract

**Background:**

Cell therapies are an emerging form of healthcare that offer significant potential to improve the practice of medicine and provide benefits to patients who currently have limited or no treatment options. Ideally, these innovative therapies can complement existing small molecule, biologic and device approaches, forming a so-called fourth pillar of medicine and allowing clinicians to identify the best treatment approach for each patient. Despite this potential, cell therapies are substantially more complex than small molecule or biologic interventions. This complexity poses challenges for scientists and firms developing cell therapies and regulators seeking to oversee this growing area of medicine.

**Results:**

In this project, we retrospectively examined the development of seven cell therapies – including three autologous interventions and four allogeneic interventions – with the aim of identifying common challenges hindering attempts to bring new cell therapies to market. We complemented this analysis with a series of qualitative interviews with experts in various aspects of cell therapy. Through our analysis, which included review of extant literature collected from company documents, newspapers, journals, analyst reports and similar sources, and analysis of the qualitative interviews, we identified several common challenges that cell therapy firms must address in both the pre- and post-market stages. Key pre-market challenges included identifying and maintaining stable funding to see firms through lengthy developmental timelines and uncertain regulatory processes. These challenges are not unique to cell therapies, of course, but the novelty of cell-based interventions complicates these efforts compared to small molecule or biologic approaches. The atypical nature of cell therapies also led to post-market difficulties, including challenges navigating the reimbursement process and convincing providers to change their treatment approaches. In addition, scaling up production, distributing cell therapies and managing the costs of production were challenges that started pre-market and continued into the post-market phase.

**Conclusions:**

Our analysis highlights several interrelated challenges hindering the development of cell therapies. Identifying strategies to address these challenges may accelerate the development and increase the impact of novel cell therapies.

## Background

Cell therapy has been defined as the therapeutic application of cells, regardless of cell type or clinical indication, used with the intentions of healing or curing a medical problem [1] and is a key component of the broader field of regenerative medicine [1, 2]. Over the last several decades, cell therapies have emerged as a novel treatment approach and met with some medical and commercial successes. By 2008, the global sales of the cell therapy industry had reached $410 million, and in 2009, it was estimated that the potential market in the United States for these therapies was more 100 million people [1].

Many medical conditions that are not well treated today occur as a result of cellular dysfunction and cell therapies, which rely on the use of cells rather than small molecules or biologics, appear to offer potential to address many of these conditions. Indeed, the use of cell therapies has been identified as a potential strategy to address a wide range of disease targets, including heart disease, diabetes, neurodegenerative diseases, musculoskeletal disorders and spinal cord injury, among others [1, 3]. While this specific list of targets may be open to debate, it is clear that scientists and clinical researchers have identified cell therapy as a promising therapeutic strategy to help patients with a broad range of medical conditions. This potential is also observed in the growth of cell therapy clinical trials with a recent study identifying 1,925 ongoing cell therapy clinical trials as of June 2010 [4]. This analysis identified a mix of autologous (using the patient’s own cells) and allogeneic (using cells from a third party) interventions and concluded that most trials were either phase 1 or phase 2 [4]. Another slightly more recent analysis of clinical trials, focusing specifically on stem cell therapies, identified trials of novel cell therapies targeting a range of different conditions and concluded that cardiovascular disease, neurological disease, cancer, liver disease and bone conditions were the most common [5].

Despite the clear potential of cell therapies, the impact of cell therapies on patients (with a few exceptions) has been limited. The difficulty successfully bringing novel cell therapies to market has been discussed in numerous commentaries on the industry and in individual discussions of unsuccessful products [6–14]. Potential explanations include the underlying complexity of cells, scalability and manufacturing concerns, and regulatory hurdles. All of these and other factors no doubt are contributors, but, to date, the translation of cell therapies has received relatively little systematic consideration. This paper attempts to contribute to our understanding of the cell therapy industry, including both its struggles and its potential. Specifically, we systematically reviewed seven cell therapy products, tracing them from early in their development to, if applicable, their market entry and market success (or failure). In addition, we complemented these case studies with a series of qualitative interviews with experts in cell therapy. We analyzed these rich qualitative data with the goal of understanding the various factors that have made it difficult to successfully develop novel cell therapies and of identifying best practices to facilitate ongoing translation and commercialization efforts.

## Methods

We selected seven cell therapy products (Epicel, Carticel, Provenge, Apligraf, Dermagraft, Osteocel, and Prochymal) that used a range of different cell types and treatment modalities and had achieved at least some modest success. For each of these products, we conducted a systematic search for documents discussing their development. Specifically, we searched Lexis-Nexis for media reports (including both mainstream and specialty sources) on each product and PubMed for scientific reports. We also reviewed company websites and, if appropriate, regulatory filings. In addition, we conducted broad-based internet searches using Google. Documents returned by these searches were reviewed to assess their relevance and the extent to which they provided novel information, duplicated or contradicted other material. Relevant documents were also used to identify additional keywords to expand our searches in an iterative manner. The goal of these searches was to identify sufficient material to develop a complete history of these products. The products we examined were primarily developed in the United States and we focus our discussion of regulatory issues on the role of the U.S. Food and Drug Administration (FDA). We cite the key sources we used to compile these histories in our results section, but we reviewed many more articles (which typically provided similar accounts) as part of our research. In all, we estimate that we reviewed more than 150 sources to assemble these seven product histories.

We selected products that had received some degree of market access to ensure our analysis did not focus solely on pre-market scientific and regulatory concerns but also examined relevant post-market considerations, such as reimbursement. One consequence of this decision was that our product histories focused on relatively early cell therapy products and do not include promising but not yet commercialized products, such as chimeric antigen receptor (CAR) T-cell therapies. We recognize that next generation cell therapy products under development today may face a different set of challenges but believe that analysis of these early products still provides valuable insight for scientists and firms developing cell therapies today.

We also conducted qualitative interviews with 12 experts in various aspects of the cell therapy industry. Interviewees were identified from media reports, conference rosters, corporate leadership, etc. and included multiple distinct perspectives on the industry. Our 12 interviewees had a variety of roles in the industry. Many were executives (ranging from VP to CEO level) at a variety of cell therapy companies. In addition, we interviewed investors, consultants and journalists that focused their efforts on the industry and academics working on the development of cell therapies. Many of our interviewees had experience in several different companies or roles relevant to the project from experiences at different companies or moves from academia to industry or vice versa. Unlike the product histories, which focused on more mature products, discussion in the interviews addressed both these mature products and new cell therapies that were still under development at the time of the interview.

To encourage our interviewees to speak candidly about their experiences, all were offered confidentiality and none are identified in the analysis. Interviews were based on a semi-structured interview guide, which ensured that key topics were discussed with each interviewee, while still allowing the interviews to flow naturally and permitting follow-up questions and discussion of topics that arose during the discussions. Each interview was recorded and professionally transcribed. The qualitative interviewing component of the project was approved by the Institutional Review Board for Human Subjects Research at Georgia Tech (Protocol H13409) and each interviewee gave their informed consent before participating in the study.

**Fig. 1:**
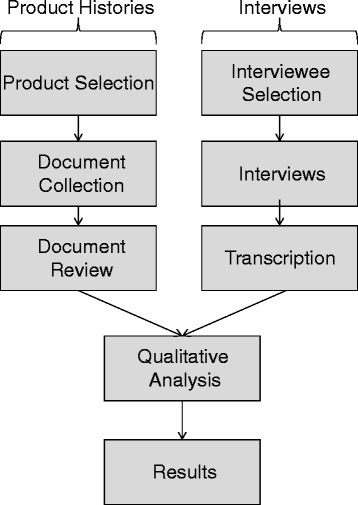
Overview of research methodology

We conducted qualitative thematic analysis of both the product histories and the interview transcripts. Specifically, both authors discussed the product histories and independently reviewed the interview transcripts. In our analysis, we used a modified grounded theory approach, relying on open coding to identify the key themes that appeared in multiple interviews. We also reviewed the themes that appeared in our analysis with concerns suggested in the extant literature. In all, our goal was to identify key challenges and best practices in the field. An overview of our methodology is provided in Fig. 1.

## Results and discussion

### Product histories for seven existing cell therapies

Despite the large number of cell therapy clinical trials [4, 5], relatively few cell therapies have made it to market in the United States. We selected seven notable products that have been successful in delivering a cell therapy to (at least some) patients for extensive analysis. The selection of these particular products was not random but purposive. We selected them because they offered a diversity of cell types, clinical modalities (i.e. autologous and allogeneic) and target medical conditions. While we cannot claim they are representative of the larger cell therapy industry (or future cell therapy products), they include many of the larger and more important contemporary cell therapies and we believe that their histories offer a perspective into the industry more broadly. An overview of these seven products is provided in Table 1 and brief product histories compiled as part of our research are provided below.

**Table 1:** Summary of cell therapies examined

Product	Current Owner	Type	Cell Source	Clinical Indication	Nature of FDA Approval(s) and year(s)
Epicel	Vericel	Autologous	Patient’s own skin	Deep dermal burns	Unregulated device (1988)
					Humanitarian use device (2007)
Carticel	Vericel	Autologous	Patient’s own cartilage	Cartilage defects	PHS Act, Section 351 (1997)
Provenge	Valeant	Autologous	Patient’s own immune cells	Advanced prostate cancer	PHS Act, Section 351 (2010)
Apligraf	Organogenesis	Allogeneic	Skin cells from human foreskin derived neonatal fibroblasts	Venous leg ulcers (VLU) and diabetic foot ulcers (DFU)	PHS Act, Section 351 (1998 for VLU, 2000 for DFU)
Dermagraft	Organogenesis	Allogeneic	Skin cells from human foreskin derived neonatal fibroblasts	Diabetic foot ulcers	PHS Act, Section 351 (2001)
Osteocel	NuVasive	Allogeneic	Mesenchymal stem cells & osteoprogenitor cells	Bone regeneration as part of spinal surgery	PHS Act, Section 361
Prochymal	Mesoblast	Allogeneic	Mesenchymal stem cells derived from adult bone marrow	Graft vs host disease	Compassionate Use (2005). Not approved for general use in US.

### Autologous cell-based therapies

#### Epicel

Epicel is a skin graft grown from a small biopsy of the patient’s healthy skin that can provide skin replacement for patients with deep dermal or full thickness burns [15]. The product grew out of the work of James Rheinwald and Howard Green, who demonstrated in 1975 that human epidermal keratinocytes could be isolated and serially cultured in vitro in their lab at the Massachusetts Institute of Technology [16]. This work with epidermal cells led to the creation of an autologous cultured skin graft and, by 1980, two patients with third degree burns had received this skin replacement therapy [17]. In summer 1983, the therapy was successfully applied on two young boys who had burns over 97 percent of their bodies [18]. The resources necessary to produce this cell therapy for human application were beyond the capacity of a university lab; thus, Dr. Green founded the company Biosurface Technology for skin graft production in 1986 [18, 19].

Biosurface Technology developed and commercialized this skin graft technology under the trade name Epicel in the mid-1980s to treat severe burn victims, and in 1988, Epicel became the first cell-based product for tissue repair commercialized in the USA [15, 20]. In 1994, Genzyme acquired Biosurface Technology [20]. Epicel was developed prior to the development of FDA regulation on cell therapies; therefore from 1988 to 1997, Epicel was used as an ‘unregulated device’ [15]. In 1998, the FDA first designated Epicel as a medical device and then, later the same year, as a humanitarian use device [15]. In 1999, Genzyme submitted a humanitarian device exemption application to the FDA and in 2007, FDA approved this application, granting Epicel market access without requiring clinical trial data demonstrating effectiveness [15].

Genzyme continued to produce Epicel until February 2011, when Sanofi bought Genzyme for more than $20 billion [21]. In April 2014, Aastrom bought Sanofi’s cell therapy and regenerative medicine portfolio and manufacturing centers for $6.5 million [22]. Aastrom which was renamed Vericel in November 2014, continues to produce Epicel and market this product worldwide.

### Carticel

Carticel is an autologous cellular product indicated for the treatment of knee cartilage defects in patients who have not responded well to previous surgical repairs [23]. The treatment involves two minor surgical procedures – the first to retrieve a tissue biopsy and the second, following cell processing, to insert the transplant. Autologous chrondocyte transplantation was pioneered in Sweden in the late 1980s and early 1990s [24, 25] and the technology was commercialized by Genzyme in the United States [23]. The regulatory approval process for Carticel was atypical but quick. Genzyme first provisionally marketed Carticel in the United States as an unregulated device in early 1995 before FDA regulations for autologous cell therapies were finalized [26]. Later in 1995, the FDA informed Genzyme that it would not regulate Carticel at the current time [27]. The FDA later reversed this decision and Genzyme responded by filing a biologics license application with the FDA for Carticel in 1996 [23]. Genzyme received accelerated approval under FDA’s new cell therapy guidelines in 1997 [23]. This approval relied on existing patient registry data, rather than a clinical trial and required that Genzyme conduct follow-up studies, including a double-blind, placebo-controlled trial to assess Carticel’s efficacy [28]. As of 2007, Genzyme reported that more than 13,000 patients had received Carticel implants in the United States [29].

Carticel has followed the same acquisition pattern as Epicel. Following its initial commercialization by Genzyme, it was produced by Sanofi from February 2011 to April 2014 and then sold to Aastrom in 2014. Aastrom was renamed Vericel in November 2014, and Vericel currently produces and markets Carticel.

#### Provenge

Provenge is approved by the FDA as an autologous cellular immunotherapy for the treatment of advanced prostate cancer [30]. The treatment involves taking white blood cells from the patient’s body, processing them in vitro to more actively attack cancer cells and then transferring them back to the patient [31]. The commercialization of Provenge dates to 1992 when Activated Cell Therapy was spun out of Stanford University [32]. By 1995, the company had been renamed Dendreon and new leadership pushed Provenge into clinical trials [32].

Dendreon continued to develop Provenge throughout the 1990s and early 2000s. Promising clinical trial data followed, but Provenge had a bumpy road to FDA approval. In 2007, a FDA advisory panel recommended its approval, but in an unusual and controversial move, the agency ignored its advisory panel’s advice and required Dendreon to provide additional evidence about the treatment [33]. Three years later, in 2010, the FDA approved Provenge under Section 351 of the Public Health Safety Act. This marked the first time a so-called “cancer vaccine” had been approved and, although Provenge offered only modest survival benefits, its approval was hailed as a major advance that might open the door to other therapies that harness the immune system to fight cancer [31].

Provenge was hailed as breakthrough for the cell therapy industry [1] but ultimately a combination of manufacturing and marketing challenges, combined with the emergence of more affordable and less complicated competition led it to underperform expectations [34, 35]. Dendreon filed for bankruptcy in late 2014 [36] and Dendreon’s assets (including Provenge) were purchased by Valeant Pharmaceuticals for $495 million in February 2015 [37]. The extent to which Valeant can turn Provenge into a commercial success remains an open question, but the company is expected to focus its efforts on cutting production costs and improving marketing to physicians [13].

### Allogeneic cell-based therapies

#### Apligraf

Apligraf is a bioengineered allogeneic skin substitute and wound healing product used to treat venous leg ulcers and diabetic foot ulcers [38, 39]. Apligraf was developed by Organogenesis, which was founded in 1985 as a spinoff from the Massachuetts Institute of Technology [40]. Appligraf received FDA approval for the treatment of venous leg ulcers in 1998 and for the approval of diabetic foot ulcers in 2000 [40].

Although Apligraf continues to be developed by Organogenesis, the company’s path and product’s finances have not been smooth. Organogenesis went public in 1986, partnered with Novartis, and ultimately filed for bankrupcty protection in 2002 [41]. These financial troubles arose in part from the high production costs of Apligraf [42]. Ultimately Organogenesis rebuffed multiple takover offers, emerged from bankrupcy as a private company, and grew Apligraf into a successful product over the next decade [41]. Apligraf has been used in more than 250,000 patients [38].

Apligraf’s commerical success has been challenged recently by reimbursement changes announced by the Center for Medicare and Medicaid Services (CMS), the federal agency that sets reimbursement levels for federally funded heatlh care and guides reimbursment decisions made by private insurers, in 2013 and implemented in 2014 [43]. This reduction in reimbursment levels has forced Organogenesis to restructure its Apligraf business, and, as of January 2014, the firm had laid off more than a quarter of its employees [44]. The ability of Apligraf and similar cell-based wound healing projects, such as Dermagraft (discussed below), to survive under this new reimbursment regime remains an open question.

#### Dermagraft

Dermagraft is a skin substitute consisting of allogeneic cells, an extracellular matrix, and a bioabsorbable mesh scaffold that is indicated to help improve closure of diabetic foot ulcers [45]. The product was first commercialized by Marrow-Tech, which was founded in 1986 [46]. The company was founded in the New York suburbs as a spinoff of work at the New York University Medical Center and Hunter College School of Health Sciences [46], but later moved to San Diego and was renamed Advanced Tissue Sciences [14].

Through the 1990s, Advanced Tissues Sciences invested more than $300 million to develop Dermagraft [14] and, in 1996, the company formed a marketing partnership with Smith & Nephew [47]. In late 2001, Advanced Tissue Sciences received approval to market Dermagraft for the treatment of diabetic foot ulcers from the FDA [14]. Despite this success, however, Advanced Tissue Sciences was unable to turn Dermagraft into a commercially successful product and filed for bankruptcy in 2002 [14]. Following this bankruptcy, control of Dermagraft was transferred to Smith & Nephew, which attempted to commercialize the therapy itself, before selling Dermagraft to Advanced BioHealing in 2006 [48]. Advanced BioHealing benefitted from an improved reimbursement environment [48] and turned Dermagraft into a successful product before Advanced BioHealing itself was bought by Shire for $750 million in 2012 [49]. Shire had hoped to make Dermagraft a key part of its new focus on regenerative medicine, but, following the reimbursement change discussed above, sold the product to Organogenesis for up to $300 million [44]. Shire did not receive any upfront payment as part of this sale and will only receive payment if Dermagraft sales meet certain targets by 2018; the firm recorded a $650 million loss on the product it had purchased only three years earlier [44].

#### Osteocel

Osteocel, now produced and marketed by NuVasive, is a cellular bone allograft that is intended for the repair, replacement, and reconstruction of skeletal defects and used primarily as an alternative to autografts – the use of the patient’s own bone – as part of spinal fusion surgery [50]. Osteocel was originally developed by Osiris Therapeutics and was launched commercially in the United States in 2005 [51]. It was the first commercial product in the United States containing allogeneic mesenchymal stem cells. Because the product was classified as a tissue transplant rather than a drug under FDA rules, it did not require pre-market approval from the agency [51].

Starting in 2005, Blackstone Medical licensed Osteocel from Osiris and distributed it under the trade name Trinity, while Osiris distributed the product under the Osteocel name [52]. In 2006, Orthofix bought Blackstone Medical for $333 million, giving it access to the Osteocel product, which it continued to market under the Trinity label [53]. This situation continued until 2008 when Osiris sold licensing rights to Osteocel to NuVasive for $35 million in upfront payments as part of a deal worth up to $137 million [54]. Orthofix was unsuccessful in its legal efforts to block this transaction and ultimately lost its ability to sell the product [53]. As of early 2015, NuVasive continues to market Osteocel and indicates that Osteocel grafts have been used in more than 140,000 patients since 2005 [50].

#### Prochymal

Prochymal, originally developed by Osiris Therapeutics, is a human mesenchymal stem cell product formulated for intravenous infusion [55]. Osiris was founded in 1992 in Cleveland to commercialize research on human mesenchymal stem cells led by Arnold Caplan at Case Western Reserve University [56]. The company later moved to Baltimore in 1995 and moved Prochymal into clinical trials for a variety of indications including graft vs. host disease (GvHD), Crohn’s Disease, chronic obstructive pulmonary disorder, diabetes and cardiac repair [53, 57]. Despite promising preclinical data, Osiris struggled to successfully bring Prochymal to market [58].

The most successful indication to date has been GvHD. Osiris received approval to offer Prochymal to a subset of patients with acute GvHD in 2005 under the FDA’s compassionate use program [59]. The company also received approval to market Prochymal in Canada and New Zealand for the treatment of acute GvHD in 2012 based on a subset of data from a larger clinical trial [60]. Canadian regulators indicated that the efficacy data were not conclusive and required Osiris to conduct a follow-up trial within five years [60].

Osiris funded its twenty-year quest to bring Prochymal to market through a variety of approaches, including venture funding, partnerships with larger pharmaceutical companies (including Novartis and Genzyme), a contract with the U.S. Department of Defense, and, in 2006, an initial public offering [55, 56, 61, 62]. In 2013, Osiris sold Prochymal to Mesoblast for up to $100 million [63]. As of early 2015, Mesoblast is continuing efforts to commercialize Prochymal for GvHD and Crohn’s Disease in both the US and, through a partnership with JCR Pharmaceuticals, the Japanese market [64].

#### Qualitative interviews with cell therapy experts

In addition to these retrospective analyses of seven cell therapy products, we conducted qualitative interviews with 12 experts in various aspects of the cell therapy industry. These interviews were organized around an interview guide to ensure each participant was given the opportunity to address a similar set of questions but ranged over a wide range of topics, reflecting each participant’s perspectives, interests, and expertise. The interviews addressed both the historical development of the cell therapy industry – including discussions of many of the seven products analyzed above – as well as ongoing preclinical and clinical research on cell therapies that have not yet made it to market.

#### Key challenges

Both our retrospective evaluations of existing products and our interviews revealed a wide range of challenges that scientists, entrepreneurs, and others working to bring cell therapies to the clinic must overcome. These challenges reflect the complicated nature of using cells as a therapeutic approach (particularly compared to small molecules or biologics), including concerns about the manufacturing of cells, as well as concerns about regulation and reimbursement.

**Fig. 2:**
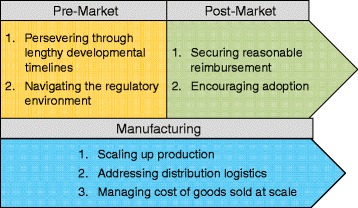
Classifications of key challenges in the development of cell therapies

In our analysis we found it useful to group these challenges into three broader categories (see Fig. 2). These three categories are “Pre-Market Challenges,” which covers pre-clinical and clinical research from the initial conception of the product idea up to the point that it gains market access, “Post-Market Challenges,” which covers the time from market access to the present (or the point when a product was no longer available), and “Manufacturing Challenges,” which covers issues associated with producing cells for therapeutic use and cuts across both pre- and post-market time periods. Some of the individual challenges that fall within each classification are shown in Fig. 2. We discuss each classification below and illustrate the challenges with key details from the product histories and quotes from the interviews.

#### Pre-market challenges

The pre-market phase has traditionally received substantial attention in discussions of the development of novel drugs and medical therapies. In particular, attention has been given to how promising new approaches supported by pre-clinical data can cross the so-called valley of death and generate sufficient evidence of safety and efficacy to attract funding to push through to commercialization [65–67]. Thus, it was not surprising that this challenge was visible in nearly all of the product histories and discussed, often in some depth, in most of our interviews. In our analysis of pre-market challenges, two main themes appeared in our research: (1) Persevering through lengthy developmental timelines, and (2) Navigating the regulatory environment. These two themes were closely related as the regulatory environment contributed to the need to persevere over long time periods in several cases. Because discussions about perseverance and long developmental timelines tended to focus on financial issues rather than interaction with regulators, however, we discuss each separately below.

##### Persevering through lengthy developmental timelines

Our retrospective product histories clearly indicated that firms should be prepared to survive for many years before successfully bringing a product to market. Osiris Therapeutics, for instance, brought Osteocel to market in 2005, 13 years after the company was founded in 1992. And Osteocel had a smoother path to market than many of the products we examined, as it did not require pre-market approval from the FDA. In contrast, Prochymal, another product developed by Osiris, only gained conditional market access in Canada and New Zealand in 2012, some 20 years after the company was founded. Similarly, Apligraf gained market access some 15 years after Organogenesis was founded and Dermagraft was granted pre-market approval 16 years after Marrow-Tech was founded.

These examples illustrate that early stage cell therapy companies must be prepared to raise sufficient capital to survive as they navigate the long and uncertain path to market. Both our product histories and our interviews highlighted this concern and the various strategies that cell therapy firms can use to try to address it. These include angel and venture funding, partnerships with larger companies and the public markets. In addition, some firms were able to develop secondary products, often with lower regulatory hurdles, to generate some positive cash flow during the pre-market period.

Several interviewees identified acquiring the funding to survive the therapy development process as the single biggest challenge facing firms in the field. One interviewee stated that “Probably the most significant challenge relates to finding appropriate financing ....” This challenge was affected by broader financial trends as well as cell therapy- or biotechnology-specific considerations. These latter issues included what one interviewee termed “a tendency to spin out ideas, patents and technologies far too early into companies,” as well as hesitancy by both venture capital and pharmaceutical firms to embrace the cell therapy space. Two quotes illustrate these latter concerns:

What has happened over the last ten years or eight years is then that the valley of death has expanded from proof of concept to filing an IND, to proof of concept to getting your phase two data completed. That has put significant financial pressure on companies….

Pharma has been absent in the cell therapy industry. That has been a huge problem…Pharma doesn’t just give you money….Having pharma come in and… partner with you validates your technology platform.

The complexities and challenges of funding early stage cell therapy companies are intensified by the reality that such funding is needed for a long but uncertain period of time and by the fact that most funding strategies available to early stage firms in the field are dilutive. Repeated use of dilutive funding mechanisms lowers the founders’ ownership stake and can, in some cases, hinder a firm’s ability to control its operations or raise additional funding.

Financing clinical development and commercialization posed a serious challenge for several of the companies developing the products we examined in our product histories and has affected other cell therapy firms as well. Because we selected our cases based on successful market entry, we have no examples of bankruptcy in the pre-market phase, but bankruptcies were seen shortly after market entry in the commercialization of Provenge, Dermagraft and Apligraf. Not surprisingly, other companies commercializing cell therapy products, including Tengion (2014), Garnet BioTherapeutics (2010) and MicroIslet (2008), have gone bankrupt during the pre-market phase [68–70].

Companies that avoid this fate may still face challenges reflecting the choices they made to acquire funding. Partnerships with pharmaceutical firms, while often a key step in the development of a successful product, can complicate operations if, for example, the cell therapy firm and its partners disagree about commercialization strategies or the most promising indications to pursue. Going public provides another source of capital for some firms but places a new set of expectations and reporting requirements on firms that may be problematic for companies in the pre-clinical and clinical research stages with little to no revenue.

##### Navigating the regulatory environment

The regulatory environment is among the many factors contributing to the need for firms developing cell therapies to persevere through a lengthy developmental timeline. Several clear themes regarding the regulatory environment for cell therapies were apparent in analysis of the interview transcripts. Most interviewees felt that when cell therapies were first emerging, the FDA struggled to regulate them, hindered in part by regulations tailored to other, very different, types of drugs. Most interviewees also indicated, however, that this situation had improved over time. Whether the current regulatory approach was working well or problematic, however, was a matter of more disagreement. Some interviewees felt that the current rules worked relatively well, despite some uncertainties and ambiguities that, at times, hindered the commercialization of cell therapies. Others believed that the agency had overreached in some of its regulatory decisions, putting an unnecessary burden on the field. The following quotes illustrate these themes:

We’re still muddling our way through the regulatory pathway for regenerative products…. Certainly FDA originally wanted to apply the same rules, and the same kinds of regulatory authority [as other products]…, but FDA has really come along. They are looking at cell therapies in the different way that they deserve to be looked at today.

The FDA’s regulatory framework that they’ve defined for cell therapies is relatively clear. The language is clear, and the framework exists, but…there’s still some ambiguity around how one interprets [it].

These comments describe an evolving but still uncertain regulatory environment. Among the key ambiguities discussed regarding the FDA regulatory approach was the distinction between a section 351 and section 361 cell therapy. Some products fall under Section 351 of the Public Health Service Act (PHS Act) while others fall under Section 361 of the PHS Act [71]. This is a key distinction because Section 351 requires pre-market approval typically via the clinical trial pathway, while Section 361 provides a shorter path to market. Some products fall clearly into one of these two categories, but others are in a gray area where the appropriate classification is not entirely certain. This risk-based regulatory approach where presumably riskier therapies required more extensive pre-market testing seemed reasonable to many interviewees.

At the same time, however, interviewees worried that the tiered approach creates several difficulties. On one hand, firms are allowed to declare that they fall under Section 361 and enter the market with reduced regulatory requirements without any formal approval from the FDA that this decision was appropriate. In this case, the firm is taking on the risk that the FDA might later indicate they were mistaken in this classification and need to conduct clinical trials and gain pre-market approval before continuing to sell their product. This situation creates uncertainty and could pose difficulties for firms attempting to raise funds. The situation also opens the door for firms to profit from selling unproven or ineffective cell therapies on the U.S. market, perhaps with the intention of shutting down when or if the FDA’s enforcement office comes calling. The growth of unproven cell or stem cell therapies has received substantial attention in recent years [72–76] and the extent to which FDA rules facilitate this practice within the U.S. raised concerns for several interviewees. Both of these concerns were exacerbated by worries that the two major criteria that the FDA uses to determine whether a cell therapy falls under section 351 or section 361 rules (“minimal manipulation” and “homologous use”) were ambiguous in at least some cases.

Those who felt that the FDA was overreaching in its oversight of cell therapy linked these concerns both to these definitions (and ultimately whether a specific product required pre-market approval) and to the agency’s broader role in the political ecosystem. As two interviewees explained:

The FDA fundamentally is a political organization and…it appears to be very concerned about its standing within the political community and has taken an overly conservative approach to the regulation of cell therapies and has made it very challenging for companies to move forward.

Definitions such as that [for minimal manipulation] have really, really not only surprised the marketplace and companies developing these cell-based products but have really been onerous in terms of what it means regarding timeline and cost to bring products forward.

As a whole, our analysis described an agency that initially struggled with cell therapies but has made substantial progress in its efforts to address this rapidly evolving technology. These efforts have not been without missteps, however, and it is clear that, at least in the views of many industry participants, the FDA has opportunities to continue to improve its regulation of novel cell therapies.

#### Post-market challenges

In addition to pre-market challenges, our research identified several post-market challenges that affected the development of cell therapies. Some of these challenges had their origins in pre-market decisions regarding research approaches or commercialization strategies but they manifested most directly after a cell therapy product had gained market access. These challenges included: (1) Securing reasonable reimbursement and (2) Encouraging adoption.

##### Securing reasonable reimbursement

Challenges securing and maintaining a reasonable reimbursement level were a frequent theme both in our product histories and interviews. The key point here is straightforward, if perhaps not as well recognized in the literature as one might hope. It doesn’t matter how good a cell therapy product is if no one is going to pay for it. And secondly, if reimbursement levels don’t at least reach and ideally exceed the cost of production, it’s going to be quite difficult to build a successful business around the product.

Interviewees expressed concern about the uncertainty regarding reimbursement decisions, particularly given the novel and expensive nature of some cell therapies and ongoing pressures to reduce health care costs. One interviewee noted that “We have inadequate precedence in the cell therapeutic space for having set price points and determining reimbursement,” a situation that makes it difficult for companies to make strategic decisions about which indications to pursue and how to optimize their operations. Another interviewee highlighted how not fully thinking through the cost-benefit calculus and its impact on reimbursement could lead to large-scale investments on cell therapies unlikely to have much clinical or market impact and ultimately lead to squandered investments in “nonviable” cell therapies.

Our product histories revealed that reimbursement was a key challenge for Dendreon as it commercialized Provenge and for both Advanced Tissue Sciences and Organogenesis as they attempted to successfully commercialize their wound healing products. Improved reimbursement was an important reason that Advanced BioHealing was able to turn Dermagraft into a successful product and helped Organogenesis as it recovered from its 2002 bankruptcy and commercialized Apligraf. After several years of market success, a reimbursement change by the U.S. Centers for Medicare and Medicaid Services (CMS) has raised questions about the viability of these products going forward, as one of our interviewees explained:

Last December, December of 2013, CMS changed all the codings for wound healing products, cutting the reimbursement by 60 percent, which resulted in Organogenesis laying off over 50 percent of their work force. Shire basically took a $650 million write-off for Advanced BioHealing and handed Advanced BioHealing for free to Organogenesis and said, “If you wanna try to fix this, fix this.” With one flick of a pen, CMS basically destroyed, once again, the two companies that were in the forefront with… cell-based products on the market.

As this case illustrates, challenges can emerge not only in the early stages of market access when a product’s initial reimbursement levels are established but later in the product life cycle, perhaps reflecting changes in the competitive environment or pressures to reduce health care costs. Although changing reimbursements levels are a challenge for many medical products, they pose particular difficulties for cell therapies with their complicated and expensive manufacturing processes.

##### Encouraging adoption

A second theme that emerged in our analysis of post-market challenges was that of promoting physician and patient adoption of new cell therapies. Both our product histories and interviews indicated that it was not enough to produce a product that was superior to its competitors and navigate the regulatory process to gain market access. Rather given the complexity of cell therapies and the differences between administering cell therapies and more traditional treatments, getting physicians to actually use a novel therapy could be quite difficult. One interviewee explicitly discussed how ease of administration could affect physicians’ treatment choices:

This is still a kind of therapy that’s difficult for most physicians, even specialists, to envision administering, and if there’s an easier way to administer almost the same kind of efficacy, they’ll jump at that every time because it’s easier to give a pill. It’s easier to give a patch. It’s easier to give even an injectable that they’re accustomed to delivering than cells because they’re different. They’re weird….They’re more finicky to administer, to manage, to process, for any of a number of reasons.

As one interviewee explained, adoption is a business model and marketing problem that requires understanding your customer and how, in particular, your product fits within the business model of the clinics you hope will use it. Ignoring (or misjudging) these issues can pose serious challenges to a company’s prospects. Reflecting on Dendreon’s attempt to commercialize Provenge, one interviewee indicated:

There’s a complication [with] which physician is actually reimbursed for the therapy. There’s sort of a patient-handling flow-chain in oncology. The company misunderstood which physician would be getting the benefits of prescribing the therapy and targeted the wrong physician population [in their marketing].

The physician adoption hurdle depends on both the nature of the therapy and its competitors in the healthcare marketplace. Autologous interventions, such as Provenge, face particular challenges because they typically require multiple coordinated interactions with the same patient, complicating the treatment process. The severity of the adoption hurdle likely also varies by indication as well as the improvement over standard of care offered by the cell therapy approach. Physicians are presumably more likely to expend the effort to master and use more complicated treatments when the benefit over the status quo is substantial. Regardless, our analysis suggested that identifying and beginning to work with the appropriate physician population early in the development of a novel cell therapy was an important best practice. This early clinician involvement can help steer companies toward products that can more easily surmount the adoption hurdle and reach patients.

#### Manufacturing challenges

In addition to the pre- and post-market challenges discussed above, it was clear in our product histories and, particularly, in our interviews that the production and distribution of cells for therapeutic purposes posed an ongoing and substantial challenge to the successful development of the cell therapy industry. This challenge reflects the greater complexity of cells compared to small molecule and biologic drugs and the diversity of therapeutic strategies (i.e. autologous and allogeneic) envisioned for cell therapies. Manufacturing issues arise early in the research process and continue through the entire lifespan of a product. Although many different aspects of manufacturing were discussed in our interviews, in our analysis we found it helpful to organize manufacturing challenges around three themes: (1) Scaling up production, (2) Addressing distribution logistics, and (3) Managing cost of goods sold. Each of these themes is discussed below.

##### Scaling up production

The challenge of scaling up production to produce a large number of cells is complicated by a variety of factors, including a lack of tools and poor scientific understanding of key issues. In addition, the scaling up process is, at times, complicated by decisions made early in the development process, when research is typically underway in an academic setting and relatively less attention is paid to the scalability of production and related costs. Moving from this academic mindset to a commercial-grade production process is complicated by the available technology and concerns that the relevant characteristics of the cells (which give them their desired activity) might be altered by changes in the production process. As one interviewee explained, this is, at least in part, an engineering issue:

There’s a whole bunch of engineering challenges involved with growing cells at large scales… You have to understand the environment that the cells are in and provide an environment as you scale up that is gonna get you the product you want. You need to understand the chemical and physical parameters that affect the cells as you go ahead and change the configuration and the scale.

Many interviewees felt that there was a greater recognition of the importance of manufacturing and the associated engineering issues in the industry today than in the past, but there were still concerns that technology was not at the appropriate point to scale and produce many cell therapies in a cost effective manner. One reason for this challenge was that limited scientific understanding hindered efforts to develop the right tests and tools to facilitate improved manufacturing of cells. One interviewee highlighted this concern by discussing the multi-mechanistic nature of some cell therapies:

There’s a lot of things the cells do, so which mechanism of action is important for efficacy, and which one should I build my potency assay around? That’s a huge challenge, because if you…make process changes…you want to make sure that it’s safe and it’s the right product…, [but] you also need to make sure it’s potent.

This quote highlights that scaling up production is not just the ability to grow cells in large numbers, using for instance, bioreactor technology, but also having the right tests and tools to ensure that cells produced through a different manufacturing process share the same characteristics as the original cells used in pre-clinical or early clinical research. This concern underlies the cell industry dogma that “the process is the product.” This statement or similar sentiment was expressed in nearly all of our interviews and, although our interviewees had mixed views on its accuracy, it was clear that uncertainty about how regulatory agencies, such as the FDA, would view process changes was a significant concern and shaped how many industry insiders thought about production and scale-up decisions.

Scaling up remains a key bottleneck in the cell therapy industry today and is important to meeting patient demand for successful therapies and for, as is discussed in more detail below, meeting this demand at a cost that still leaves room for a firm to profit within the current healthcare marketplace.

##### Addressing distribution logistics

Cell therapies are promising because they contain living cells, but the living nature of cell therapies greatly complicates the process of getting these treatments to patients. Most cell therapies will not remain viable at ambient temperature over an extended period of time and will, thus, require more complicated distribution strategies than typical small molecule therapeutics. These distribution challenges apply to both allogeneic and autologous cell therapies but are typically more acute for autologous interventions, where the products often involve moving patient cells to the processing facility and sending the processed therapeutic cells back to the patient’s physician for treatment. The extent to which a cell therapy can be frozen and thawed and maintain its activity impacts distribution decisions as does the length of the product’s stability at various temperatures. Although both pose challenges, a product with a shelf life of only two days poses substantially greater logistical issues than a product that remains stable for a week. One interviewee described the challenge for an autologous therapy as follows:

Often you procure starting material from a patient…It’s shipped to a manufacturing site. Manufacturing is conducted under GMP and then final product is shipped back to that patient. Getting the right product back to the right patient is important issue. Keeping control of that cold chain during the procurement and the distribution is…[a]complicated issue with cell therapies, which often have…a very short shelf life.

Firms are exploring various models to tackle these distribution challenges, including shipping products both frozen and thawed. In addition, some firms are distributing their manufacturing facilities to ensure that production occurs closer to patients and, thus, reduce transit times. As one interviewee articulated, addressing these logistics is still a work in progress:

Some companies are working on having a cryopreserved supply chain. Other companies are thawing the product onsite and then reconstituting into a new formulation… and they’re getting stability of two to four days, which is enough time to ship, but then you’re talking about just in time shipping for your product commercially, which is a very big challenge. Neither one is ideal.

The technical difficulties that affect scale up efforts also complicate distribution. In the absence of a good potency assay, for example, it’s hard to know how distribution processes are affecting a cell therapy product. As one interviewee explained, “Your product may be going to hell, but you don’t really even know it. It’s a big challenge right now that people haven’t really sorted out yet.”

Sorting out distribution logistics is an important issue for several reasons. It may be the case, for example, that a cell therapy is effective in an early stage clinical trial when manufacturing occurs on-site and distribution is not required, but that distribution issues leave the product less effective or wholly ineffective. In addition, if some non-trivial portion of the product is lost due to distribution issues, this could complicate the physician adoption concerns discussed previously and increase the cost of the therapy, hindering commercialization efforts.

##### Managing cost of goods sold

Both scale up and distribution contribute to the what many interviewees viewed as the key manufacturing issue – keeping costs low enough that a therapy could prove a commercial success. While the ultimate financial success of a cell therapy product depends both on price and cost considerations, cost was viewed as largely under the control of the firm producing the therapy while pricing reflected the (sometimes unpredictable) reimbursement decisions of government bureaucrats as well as the competitive environment. Several interviewees highlighted the importance of thinking about the cost of production early on in the development process, as the following quote illustrates:

A lot of people in cell therapy haven’t really appreciated that by engineering your manufacturing process early on, you can help to reduce those costs….If you can make the best cell in the world, but it costs more than you can actually sell it for, than you’re not gonna ever commercialize that. It’s never gonna have any impact on patients....

These concerns about optimizing manufacturing too late highlight the financial conundrum firms face as they scale up production and try to reduce their costs of goods. Such choices involve substantial upfront investment in equipment, such as bioreactors, as well as, in some cases, specialized GMP production facilities. In some cases, a company can’t supply the market without these facilities, but if the facilities are overbuilt or completed too soon, a company may not be able to afford them and such investments can burden a firm and leave it in dire financial straits. Indeed, one reason for Dendreon’s struggles in its efforts to commercialize Provenge was the debt it assumed, in part, to finance its production facilities. This investment proved detrimental to the company when Provenge approval and adoption lagged and the company was left with expensive but underutilized production facilities. As one interviewee noted, similar missteps were visible earlier in the history of cell therapies:

What came to mind for me is didn’t [Dendreon] learn anything from Advanced Tissue Sciences? ATS was the poster child for the field back in 2000….They were the biggest and most valuable company in the field. They had a market cap of over $1 billion in 2000. They raised in excess of $500 million and built this wonderful facility in Southern California where they were going to…engineer all parts of the human body and got out a little bit ahead of the market, spent a lot of money on this manufacturing facility and it ultimately buried them.

It is, of course, easy to diagnose these sorts of missteps in retrospect. At the time, however, firms were working based on incomplete information and projections of future sales and trying to thread the needle between overbuilding their production facilities and having insufficient supply to meet demand for an otherwise profitable product. There is no clear right answer in these situations, but these cases suggest that erring on the side of caution and growing at a sustainable rate rather than racing to meet potential but uncertain market demand may be a safer choice. Multiple interviewees suggested that operating in a capital efficient manner as much as possible was critical to success in this field and some suggested that partnering with contract research organizations and contract manufacturing organizations later in the research and manufacturing process, while delaying investment in large production facilities, might be a wise strategic choice for some companies commercializing cell therapies.

#### Interactions among key challenges

Each of the individual challenges discussed above complicates the development and commercialization of cell therapies and each challenge is enough, in certain circumstances, to push a cell therapy from promising to non-viable commercially. A striking feature of both our analyses, however, was the extent to which these various challenges interacted. It’s not enough to simply address one issue to bring a successful cell therapy to market. Rather numerous challenges – including those associated with the scientific aspects of cell therapies, as well as financial and regulatory aspects – must be addressed nearly simultaneously and with the understanding that actions taken to address one challenge may potentially exacerbate other challenges. Several key interactions identified in our analysis are discussed below, although, due to space considerations, not all possible interactions are addressed.

The “the process is the product” dogma discussed previously perhaps best illustrates these interacting challenges. Firms understand the need to improve their manufacturing processes and reduce costs yet are nervous to alter their production processes, lest they draw unnecessary attention from regulators and delay their clinical research or commercialization prospects. This situation is further exacerbated by the difficulty many cell therapy firms have acquiring sufficient capital and the worry that a regulatory delay may lead them to run out of resources. Interviewees had mixed views on the difficulty of changing a production process mid-development, but agreed that concerns and uncertainty about these sorts of changes were hindering the field. As one interviewee noted, the perception of process lock-in matters, even if regulators would allow changes to be made, particularly when time and funding pressures are considered:

Probably the most legitimate criticism that has been leveled at the FDA regarding the development of these and other technologies…is the extent to which they lock people into a specific process….[But] the truth is you can change your process from phase one to phase two. There are comparability studies…you can do to demonstrate to the FDA that your end product, your release criteria are the same, but sometimes, people don’t quite get that done in the rush to get through the phase three trial because they’re facing a cash-out deadline and they have limited resources. This all ties back to the limited financing available.

The financial challenges that cell therapy firms face are also exacerbated by both the scientific and policy challenges associated with the industry. In an industry with few successful products and no real blockbusters, investors are understandably hesitant, but, as the quote below illustrates, this risk-aversion can be reinforced and intensified by concerns over the policy environment:

I think that investors would be far more willing to invest if they knew what the rules were….When the FDA and when reimbursement can change the playing field overnight [in cell therapy], investors just get leery. They are once again leery that this is just gonna be too complicated a set of products to really ever make tremendous profit.

These interactions extend to other challenges as well. Concerns about the business model, for instance, may hinder access to funding just as much as regulatory issues and financial limitations may push firms to make poor business decisions. One interviewee attempted to describe the difficulties associated with developing cell therapies specifically by focusing on these interactions:

There are scientific hurdles. There are technical hurdles from the manufacturing side. There are logistical hurdles about getting these cells into the clinic without people screwing them up before they actually make it into the patient. There are regulatory hurdles to actually run the right clinical trial, and be addressing the right patient populations. There are business hurdles for building a sustainable business once you’ve got all those other things worked out. They’re all intertwined. It’s not like one just follows before the next. They’re all being, basically, pushed in parallel, and it takes a huge organization with extremely deep pockets to be able to address each of them sufficiently.

Needless to say, the development of most novel cell therapies is not led by huge organizations with extremely deep pockets. As a result, the early history of cell therapies, as seen in our product histories and discussed by our interviewees, is filled with examples of one or more of these interacting challenges tripping companies up and derailing or at least delaying the development of successful products. That said, some cell therapies have successfully made it to market, helping patients and offering financial rewards to at least some of the scientists and investors involved in their development.

## Conclusions

This article has focused on understanding the various challenges that scientists and firms face as they attempt to develop new cell therapies and bring them successfully to market. (See Table 2 for an overview of key conclusions.) There is no doubt that this is a complicated and difficult process with many potential pitfalls. Not only is the science of cell therapies difficult, but acquiring sufficient funding and navigating an improving but still complicated and, at times, uncertain regulatory environment is difficult. In addition, even when market access is achieved, acquiring and maintaining sufficient reimbursement is far from certain, particularly given pressures on broader health care costs, and physician adoption cannot be assumed. Furthermore, ever when these hurdles can be surmounted, firms face challenging manufacturing issues that may compromise profitability and limit the ability of a firm to survive.

**Table 2:** Summary of key conclusions

Key Challenges	
• Pre-market: Persevering through lengthy developmental timelines and navigating the regulatory environment	
• Post-market: Securing reasonable reimbursement and encouraging adoption	
• Manufacturing: Scaling up production, addressing distribution logistics and managing cost of goods sold	
• Interactions among these various challenges complicate the process of commercializing cell therapies	
Preliminary Best Practices	Policy Considerations
• Preparing for commercialization early in the development process	• Improving coordination and communication between relevant agencies
• Adopting strategies to use capital efficiently	• Promoting international harmonization of cell therapy regulations

Although this article has focused on the difficulties associated with developing cell therapies, it would be inappropriate not to mention the positives. Nearly all of our interviewees were extremely optimistic about the future of cell therapies and believed that although the challenges were large, they would, with time, be overcome. Indeed, this article focused on the difficulties that accompany the development of novel cell therapies to help serve as a guide for scientists, firms, and policymakers considering how best to facilitate the ongoing and future development of cell therapies.

Our analysis of the challenges associated with the development of cell therapies also suggests some preliminary best practices for scientists, firms, and regulators to consider. The most straightforward recommendation arising from this research would be to focus on commercialization early. This recommendation reflects the reality that seemingly innocuous decisions made early (perhaps in an academic research environment) in the development process can greatly complicate later translation and commercialization efforts. This recommendation involves not just forethought regarding the future manufacturing processes to be used in the production of a cell therapy but also proactively identifying and engaging the relevant physician community to facilitate later uptake of the therapy.

A second best practice would involve focusing on the efficient use of capital. This might entail outsourcing and collaboration as well as careful stepwise expansions to balance the need to meet market demand and investor expectations with a reasonable cost basis. An alternative approach that was helpful for some firms was to develop a secondary product line that was subject to less stringent regulatory hurdles and, thus, had an easier path to market. In some cases, this strategy provided a positive cash flow to help a company survive the lengthy developmental timeline before their primary product could make it to market. Of course, this recommendation only applies to certain firms (depending largely on the area of their core interest and type of intervention they are planning) and must be carefully evaluated, as it runs the risk of drawing focus away from a firm’s key product and ultimately proving counter-productive.

Our study of the challenges associated with bringing cell therapies to market also offers lessons for government officials working to oversee the development of and determine reimbursement levels for novel cell therapies. Our research clearly illustrates the challenge facing regulators, such as the FDA. On one hand, each cell therapy is novel and distinct and firms need the FDA to recognize this and evaluate potential therapies using approaches that reflect their unique characteristics and mechanisms. On the other hand, scientists and firms commercializing cell therapies need consistency to inform their strategic decision-making and help them make wise development choices. This is not an easy balance for regulators to navigate, and our research suggests that regulation is improving, at least in the case of the FDA, but that more can certainly be done. Recent efforts to clarify the “minimal manipulation” and “homologous use” rules that play a role in determining whether a cell therapy requires pre-market review will hopefully prove, with time, to be a step in the right direction, but the FDA should continue to pursue opportunities to clarify its rules, particularly when it can do so without compromising its ability to tailor regulatory requirements, such as the specific evidence required to approve an IND request, to individual therapies.

Our analysis also suggests that greater attention could be given to harmonization among government agencies in a single country, such as the United States, as well as to differences between regulators in different countries. Limited coordination regarding the regulation and reimbursement of novel cell therapies within individual countries, such as the United States, has, for example, complicated the development of some products and the regulatory patchwork of differences from one country to the next is forcing companies to make difficult strategic decisions about where to commercialize their products.

Ultimately our work highlights the many difficulties that scientists and firms must face as they work to bring cell therapies to market. Despite these challenges, many are persevering in their efforts to develop novel cell therapies and improve patient outcomes for a variety of medical conditions. We hope the analysis presented here offers some useful lessons for the current generation of cell therapy innovators and helps some of these therapies make their way successfully to patients.
